# Risk Assessment of Patients After ST-Segment Elevation Myocardial Infarction by Killip Classification: An Institutional Experience

**DOI:** 10.7759/cureus.12209

**Published:** 2020-12-21

**Authors:** Kashif A Hashmi, Fahar Adnan, Omer Ahmed, Syed Rafay Yaqeen, Javaria Ali, Muhammad Irfan, Muhammad M. Edhi, Atif A Hashmi

**Affiliations:** 1 Cardiology, Chaudhry Pervaiz Elahi Institute of Cardiology, Multan, PAK; 2 Cardiology, Jinnah Hospital, Lahore, PAK; 3 Internal Medicine, Liaquat National Hospital and Medical College, Karachi, PAK; 4 Internal Medicine, Baqai Medical University, Karachi, PAK; 5 Pathology, Liaquat National Hospital and Medical College, Karachi, PAK; 6 Statistics, Liaquat National Hospital and Medical College, Karachi, PAK; 7 Neuroscience/Neurosurgery, Rhode Island Hospital, Warren Alpert Medical School of Brown University, Providence, USA

**Keywords:** st-segment elevation myocardial infarction, heart failure, in-hospital mortality, killip class

## Abstract

Introduction

The Killip classification system was introduced for clinical assessment of patients with acute myocardial infarction (MI). It stratifies individuals according to the severity of their post-MI heart failure. This system provides effective stratification of long-term and short-term outcomes in patients with acute MI and influences the treatment strategies. Revalidation of Killip class in our local population is mandatory. We planned this study to increase cardiologist's readiness to tackle the risks associated with increased mortality in each class post ST-segment elevation MI (STEMI). Objectives were to determine the frequency of Killip classes I, II, III, and IV and in-hospital mortality in each Killip class in patients with left ventricular failure secondary to STEMI.

Methods

A retrospective cross-sectional study was conducted in the Department of Cardiology, Jinnah Hospital, Lahore, over a period of three years. Patients with STEMI were stratified using Killip classification, and validation was performed by determining the within 15 days in-hospital mortality in each Killip class.

Results

The frequency (percentage) of patients with STEMI in each Killip class from I to IV was 395 (81.4%), 46 (9.5%), 27 (5.6%), and 17 (3.5%), respectively, while the in-hospital mortality in each Killip class came out to be 39 (9.9%), 4 (8.7%), 25 (92.6%) and 17 (100%), respectively. The presence of diabetes, history of smoking, and body mass index (BMI) of more than 30 kg/m^2 ^were significant contributors to mortality, along with higher Killip class and age of presentation.

Conclusions

It is concluded that the Killip classification system is a valid tool for risk stratification for patients after STEMI, especially in resource-limited countries.

## Introduction

Myocardial infarction (MI) serves as a common entity among the life-threatening diagnoses in emergency hospital admissions. The first few hours are critical as most of the complications occur during this time [1]. With the advent of new biomarkers, guideline committees have upgraded the criteria for diagnosis and inclusivity of suspected cases [2]. As such, it has greatly improved the prognosis of patients with left ventricular systolic dysfunction (LVSD) or heart failure (HF) [3].

The Killip classification was introduced for clinical assessment of patients with acute MI, and it stratifies individuals according to the severity of their post-MI HF [4]. This system provides effective stratification of long-term and short-term outcomes in patients with acute MI and influences the treatment strategies [5]. While evaluating post-MI HF, it is important to understand that these patients have a complex clinical syndrome in which increased myocardial damage or stress initiates a systemic response that provides short-term support to the cardiovascular system while also adversely affecting its structure and function. The goals of clinical assessment are to assess the severity of the disease and predict prognosis [6]. The Killip classification serves as an independent predictor of early mortality after MI, and the presence of LVSD (ejection fraction < 50%) and high Killip class predicts poor short-term prognosis [5]. In a study, the in-hospital mortality rates for ST-segment elevation MI (STEMI) were 2.9%,13.6%, 27.4%, 50.5%, respectively, for Killip classes I, II, III, IV. Out of 19,158 cases, 6,059 (31.6%) belong to class I, 978 (5.1%) belong to class II, 320 (1.6%) belong to class III, and 69 (0.36%) belong to class IV [7].

The rationale of our study was that early diagnosis of high-risk patients might help reduce the mortality associated with MI. The above-cited studies emphasize the continuous use of Killip class in a resource-depleted setting like ours and still find its utility in risk stratification of patients with left ventricular failure (LVF) secondary to MI. MI is associated with high mortality and multiple complications in our population [8,9]. As no advanced facilities (emergency angioplasty and emergency bypass surgery) are available in most tertiary care hospitals, mortality may be different in each class. Revalidation of this classification in the Pakistani population will help reduce the burden on units and increase a cardiologist’s readiness to tackle the risks associated with increased mortality in each class. This article was previously published as a preprint in Research Square (Preprint: Hashmi KA, Adnan F, Hashmi AA, Ali JP, Irfan M, Khan A, Edhi MM. Risk Assessment of Patients After ST-Segment Elevation Myocardial Infarction by Killip Classification: An Institutional Experience; 2020).

## Materials and methods

A retrospective cross-sectional study was conducted in the Department of Cardiology, Jinnah Hospital, Lahore, Pakistan, over a period of three years. A total of 485 patients presenting with STEMI admitted via emergency, fulfilling the inclusion and exclusion criteria, were enrolled in the study. The estimated sample size was 485 patients at 95% confidence level, 1.5% margin of error, taking an expected percentage of patients with in-hospital mortality in Killip class I to be 2.9% [7]. Patients of either gender with LVF secondary to STEMI and age 40 to 70 years were included in the study. Patients with acute or chronic liver disease, auto-immune disease, and diagnosed cases of chronic obstructive pulmonary disease and chronic kidney disease (serum creatinine > 1.4 mg/dL) determined by history and laboratory examination were excluded from the study. Additionally, patients with incomplete hospital records were excluded from the study. All patients were treated according to the department protocols and were classified according to the Killip classification. Killip classes were defined as given below [10]:

• Killip class I: patients without any clinical sign of HF

• Killip class II: patients with crackles or rales in the lungs, elevated jugular venous pressure, and an S3 gallop

• Killip class III: patients with evident acute pulmonary edema

• Killip class IV: patients with cardiogenic shock or hypotension (systolic blood pressure < 90 mmHg) and features of low cardiac output (oliguria, cyanosis, or impaired mental status)

The history of diabetes, smoking, and body mass index (BMI) > 30 kg/m^2^ were treated as effect modifiers. A proforma for assessing patient’s demographics was filled for each patient from hospital archives. The follow-up period for each patient was 15 days during his/her stay at the hospital for assessing prognosis.

Data analysis was performed using Statistical Package for Social Sciences (Version 26.0; IBM Inc., Armonk, USA). Numerical variables like age were described as means and standard deviations, while categorical variables like Killip class, history of diabetes, smoking, BMI > 30 kg/m^2,^ and in-hospital mortality were described as frequencies and percentages. Data were cross-tabulated, and post-stratification chi-square and Fisher exact tests were applied. Independent sample t-test and analysis of variance (ANOVA) were used to determine the mean age distribution across the in-hospital mortality and Killip classes. P-values ≤ 0.05 were considered significant.

## Results

The mean age of the patients was 57.57 ± 6.43 years; 25.4% of the patients were female, while 74.6% were male. Of all, 259 (53.4%) patients were diabetic, 196 (40.4%) patients were current smokers, and 44.7% had a BMI greater than 30 kg/m^2^. Most of the patients were in the Killip class I (81.4%), and least were present in the Killip class IV, i.e., 3.5%. A total of 85 patients (17.5%) died during their hospital stay within 15 days of MI. Descriptive statistics of the study population are presented in Table 1.

**Table 1:** Descriptive statistics of the studied population * Mean±SD (standard deviation)

Clinical characteristics		Frequency (%)
Age (years)*		57.57±6.43
Gender	Male	362(74.6)
	Female	123(25.4)
Diabetes mellitus	Yes	259(53.4)
	No	226(46.6)
Smoking	Yes	196(40.4)
	No	289(59.6)
Obesity	Yes	217(44.7)
	No	268(55.3)
In-hospital mortality	Yes	85(17.5)
	No	400(82.5)
Killip class	Class I	395(81.4)
	Class II	46(9.5)
	Class III	27(5.6)
	Class IV	17(3.5)

When Killip classes were cross-tabulated with the in-hospital mortality, there was a significant difference when Fisher exact test was applied (p-value < 0.01). When Killip classes were cross-tabulated against gender, there was an insignificant difference. The frequency of diabetes was significantly high in Killip class III. Similarly, the frequency of smoking and BMI greater than 30 kg/m^2^ were significantly higher in the Killip classes II-IV. Mean comparison and association of Killip classes according to population characteristics are presented in Table 2.

**Table 2:** Association of Killip class with clinical characteristics of the studied population * Chi-square test was applied. ** Fisher exact test was applied. *** Analysis of variance (ANOVA) was applied.

Clinical characteristics		Frequency (%)				P-value
		Class I	Class II	Class III	Class IV	
Age (years)***		56.27±5.98	62.43±6.84	64.89±1.34	63.12±0.33	<0.01
Gender*	Male	296(74.9)	32(69.6)	22(81.5)	12(70.6)	0.690
	Female	99(25.1)	14(30.4)	5(18.5)	5(29.4)	
Diabetes mellitus*	Yes	215(54.4)	17(36.9)	20(74)	7(41.2)	<0.01
	No	180(45.6)	29(63.1)	7(26)	10(58.8)	
Smoking**	Yes	111(28.1)	42(91.3)	26(96.3)	17(100)	<0.01
	No	284(71.9)	4(8.7)	1(3.7)	0(0)	
Obesity**	Yes	132(33.4)	42(91.3)	26(96.3)	17(100)	<0.01
	No	263(66.6)	4(8.7)	1(3.7)	0(0)	
In-hospital mortality**	Yes	39(9.9)	4(8.7)	25(92.6)	17(100)	<0.01
	No	356(90.1)	42(91.3)	2(7.4)	0(0)	

In-hospital mortality was found to be higher in males than in females (p = 0.01). Similarly, in-hospital mortality was higher among diabetics, current smokers, and BMI > 30 kg/m^2^. When the independent sample t-test was applied, older patients were more associated with mortality. Mean comparison and association of in-hospital mortality with population characteristics are presented in Table 3.

**Table 3:** Association of in-hospital mortality with clinical characteristics of the studied population * Chi-square test was applied. ** Independent t-test was applied.

Clinical characteristics		In-hospital mortality frequency (%)		P-value
		Yes	No	
Age (years)**		62.55±5.25	56.51±6.15	<0.001
Gender*	Male	72(84.7)	290(72.5)	<0.01
	Female	13(15.3)	110(27.5)	
Diabetes mellitus*	Yes	56(65.9)	203(50.8)	<0.01
	No	29(34.1)	197(49.2)	
Smoking*	Yes	80(94.1)	116(29)	<0.01
	No	5(5.9)	284(71)	
Obesity*	Yes	72(84.7)	145(36.3)	<0.01
	No	13(15.3)	255(63.7)	

## Discussion

In this study, we found that in-hospital mortality was higher in Killip classes III and IV than Killip classes I and II. Moreover, in addition to age and male gender, diabetes, obesity, and smoking were significantly associated with in-hospital mortality.

Over time, there has been a significant improvement in the diagnosis and management of complications of MI [11,12]. However, Pakistan is a resource-limited country with a lack of well-developed screening programs due to which patients present late, leading to a high rate of complications, like left ventricular thrombosis [13]. Moreover, the frequency of multi-vessel disease, even in the absence of ST-segment elevation on the electrocardiogram, is high [14].

The frequency of patients with post-MI HF decreased from Killip classes I-IV. Of all, 395 (81.4%) individuals fell in Killip class I, 46 (9.5%) in Killip class II, 27 (5.6%) in Killip class III, and 17 (3.5%) in Killip class IV. Also, 85% (17.5%) died during their stay in the hospital. It is high compared to developed nations, owing to delayed presentation and poor patient compliance.

Our results showed that diabetes and smoking were significantly associated with MI. Coronary heart disease was common among obese patients, as our study depicted that 44.7% had a BMI > 30 kg/m^2^. Killip class significantly determined increased mortality in our sampled population.

Our results are concordant with the published literature. Mello et al. [15] conducted a study in Brazil, where they evaluated 1,906 patients with documented MI with a mean follow-up of five years for assessing mortality. They developed Kaplan-Meier (KM) curves for comparison between survival distributions according to Killip class. Their results showed that the Killip classification played a relevant prognostic role in mortality at a mean follow-up of five years post-MI. Similarly, a study by Khot et al. [16] also showed that higher Killip class was associated with higher mortality at 30 days.

One of the limitations of the study was that it was a single-center study; therefore, we recommend large-scale re-validation of our findings in a multi-institutional study. Second, our follow-up period was short, i.e., 15 days. Moreover, the small sample size is yet another limitation of our study. 

## Conclusions

It is concluded that Killip classification is a valid tool for risk stratification of patients with STEMI, especially in resource-limited countries. Killip classification has proven to be a good, reliable, and valid tool for early risk stratification of patients with MI. Almost 100% mortality was found in Killip class IV in our study. The presence of diabetes, smoking, and obesity increased the likelihood of increased risk and higher Killip class, as shown by our results.
